# Clinical and Ultrasonographic Characterization of Hidradenitis Suppurativa in Female Patients: Impact of Early Recognition of the Disease

**DOI:** 10.3390/life13081630

**Published:** 2023-07-27

**Authors:** Antonella Di Cesare, Elia Rosi, Paolo Amerio, Francesca Prignano

**Affiliations:** 1 Section of Dermatology, Department of Health Sciences, University of Florence, 50122 Florence, Italy; antonelladicesare@yahoo.it (A.D.C.); elia.rosi@unifi.it (E.R.); 2 Dermatologic Clinic, Department of Medicine and Ageing Science, “G. D’Annunzio” University, 66100 Chieti, Italy; p.amerio@unich.it

**Keywords:** hidradenitis suppurativa, genital, ultrasound, early onset, female

## Abstract

Hidradenitis suppurativa (HS) is considered a post-pubertal disease; however, earlier onset is not infrequent. The burden of HS on the female population is very relevant, and early identification might reduce the quality of life impairment and improve the therapeutic approach. In this study, we investigated clinical biomarkers of HS that could impact the management of patients affected by HS. Female patients affected by stable HS were prospectively included in this study. Anamnestic data, clinical and ultrasonographic features were collected and analyzed. Overall, 53 patients were included in this study. The median age of onset was 19 (IQR: 14–25). Early onset was reported by 22/53 patients (median age of onset: 14; IQR: 11–16). Four patients had pre-menstruation occurrence. Early-onset patients had an earlier first-menstrual-cycle age and more frequent genital localization of HS, and were more often treated with biologics. Patients with early-onset-HS and genital localization had more severe disease with a higher number of areas affected, Hurley, and IHS4 scores. Genital involvement might be prevalent in patients with early-onset HS, leading to a worse impact on the global severity of the disease and tailored treatment protocols, including multidisciplinary approaches, in order to improve the early recognition of hidden lesions.

## 1. Introduction

Hidradenitis suppurativa (HS) is a chronic recurrent cutaneous disease that affects the terminal hair follicle in apocrine-gland-rich areas of the body [1]. The exact prevalence is not really known, ranging from 0.0003% to 4% [2,3,4], according to different studies, different geographies and ethnicities, and also to often-underdiagnosed diseases. This may reflect the diverse proportion of female and male patients across different countries, as well. Female-to-male ratios of 3:1 and 1:2 have been reported in North American and European versus South Korean patients, respectively [2,3,4]. 

Complex external environmental and lifestyle factors behave as triggering agents in genetically predisposed patients. A positive family history has been observed in approximately one-third of patients with HS [1,2,3,4]. Deep-seated, painful nodules, abscesses, and tunnels, associated with itching, burning, and malodor with functional impairment due either to disease activity or tissue fibrosis/post-surgical scarring are the typical elementary lesions of HS. Disease severity is variable among patients, from a few lesions to disfiguring manifestations; moreover, the disease course is unpredictable and, not infrequently, could rapidly evolve. HS is associated with multiple comorbidities, from general inflammatory and immune-mediated conditions (such as metabolic syndrome, including type II diabetes, or inflammatory bowel disease, IBD) to work disability, poor quality of life, and psychological comorbidities, including depression, anxiety, and social stigma [1,4,5]. It has been reported that patients with HS have greater feelings of rejection, shame, anxiety, and embarrassment, resulting in a significant lack of self-confidence, social isolation, and even depression, as well as lower perceived attractiveness than healthy people [6]. 

This is notable, if we consider that HS typically affects patients in their second and third decades of life. Furthermore, a long delay of up to 7 years is commonly observed before the proper recognition of the disease, leading to disease progression, undertreatment, and high psychosocial burden [7]. A younger age of onset has been reported in 2.2% up to 38.3% of cases; however, the definition of early onset is not consistent, as different authors have considered as criteria, for instance, age below 16 years, age below the mean-age/country of adrenarche onset, or the age of occurrence of adrenarche [2,8,9].

Data on risk factors for the early development of HS are conflicting. Some authors claim that positive family history, female sex, and presence of elements of the follicular occlusion tetrad, such as acne conglobate and pilonidal sinus, increase the risk of early onset [8,9,10], while others have found that males are more likely to experience prepubescent onset of hidradenitis suppurativa [11,12]. 

However, generally, authors agree that occurrence in childhood requires special care, including hormonal dysregulation, premature adrenarche, and therapeutic challenge [13,14].

No differences in the severity of HS in adult life for patients with early- versus adult-onset HS have been reported so far, in terms of the Hurley stage, International Hidradenitis Suppurativa Severity Score System 4 (IHS4), or the number of body areas involved in HS [9,11], although these patients might have a more disseminated disease, a longer disease duration, and a perception of more severe manifestations [15].

The treatment of HS is challenging as well, in terms of both the prevention of disease progression and therapeutic effectiveness. Antibiotics delivered topically and systemically (rifampicin and/or tetracyclins) are the mainstays, mostly due to their anti-inflammatory effect. Biologic agents (i.e., adalimumab, which is the only approved molecule so far) play a role in disease stabilization and disease modification. Medical therapies could be used alone or in combination with surgery, either to reduce inflammation and lesion extension in preoperative settings or to maintain postsurgical results, overall improving the patient’s quality of life [16,17,18].

In this context, we investigated the clinical and ultrasonographic features in a female population of patients affected by HS in order to improve the earlier recognition of the disease, providing clinical features that could be investigated, also in a multidisciplinary team, and that could lead to timely and effective treatment protocols.

## 2. Patients and Methods

All female patients affected by stable HS, diagnosed according to Dessau criteria [1], who were referred to our Outpatient Clinic for HS in Florence, Italy, during the last 5 years were prospectively enrolled in the study. The age at observation, age at onset, age at diagnosis, smoking habits, age of first menstrual cycle occurrence, menses abnormalities (from irregular menses to polycystic ovarian syndrome, PCOS, precocious puberty, and endometriosis), family history of HS, personal history of acne and other comorbidities (e.g., pilonidal cyst, IBD, and metabolic syndrome), BMI (body mass index), clinical data (site of onset and area involved in clinical observations, type and number of lesions, and Hurley and IHS4 scores), ultrasonographic data (including the clinical–sonographic scoring system, SOS-HS score), and previous and current topical and systemic therapies or surgery were recorded for each patient.

Each patient included in the study was also studied with high-frequency, 16–18 mHz, ultrasound (esaote, MyLabTouch, variable-frequency probe 12–18 mHz), and staged according to SOS-HS. All the affected body areas were scanned and the results were recorded in order to better assess disease severity, to calculate the IHS4 score, and to perform follow-up treatments. 

Early-onset HS was defined as HS with onset below the age of 18 years. Puberty was defined as the occurrence of the first menstrual cycle. The term genital area was used to refer to the pubic, vulvar, perineal, and perianal locations. Informed consent was obtained from each participant in the study; the study protocol was approved by the Local Ethics Committee (CEAVC 19799).

Descriptive statistics were used to summarize the characteristics of the study population. For continuous variables, the significance of the difference between the medians of the groups was investigated by using the Mann–Whitney test. Fisher’s exact test and the chi-square test for trends were used to evaluate categorical variables. Statistical significance was set at *p* < 0.05. Analysis was performed using GraphPad Software, by Dotmatics.

## 3. Results

### 3.1. Total Population

The baseline features of the total population included in this study are listed in Table 1.

Overall, 53 female patients affected by HS were included in the study. The median age at visit was 30 years (IQR:22–43), while the median age at onset was 19 years (IQR:14–25), with a median diagnostic delay of 3.5 (IQR:1–9). In 27/53 (50.9%) patients, diagnostic delay was >2 years; in 18/53 (33.96%), it was >5 years; and in 11/53 (20.8%), it was >10 years.

Almost 60% (31/53) of the patients were overweight (BMI > 25 kg/m^2^), while 20.8% of the patients (11/53) were severely obese (BMI > 30 kg/m^2^). The patients were active or past smokers in 67.9% of cases (36/53), while 7/53 (13.2%) had a positive family history of HS and 15/53 (28.3%) had a personal history of acne. Twenty-eight out of fifty-three (52.8%) patients reported comorbidities other than acne or menstrual cycles abnormalities, including pilonidal cysts (5/53 patients, 9.4%), type II diabetes (4/53 patients, 7.5%), and allergic status (4/53 patients, 7.5%). 

Disease severity was assessed according to the Hurley and IHS4 scores. A homogeneous distribution among different Hurley subtypes and IHS4 scores was found (Table 2). 

Patients were classified as Hurley I in 18/53 (33.9%) cases, Hurley II in 21/53 (39.6%) cases, and Hurley III in 14/53 (26.4%) cases. IHS4 had a median of 6 points (IQR 3.3–11.8) and was distributed as follows: (i) ≤3 in 14/53 patients (26.4%); (ii) 4–10 in 23/53 (43.4%) patients; and (iii) ≥11 in 16/53 patients (30.2%).

**Figure 1:**
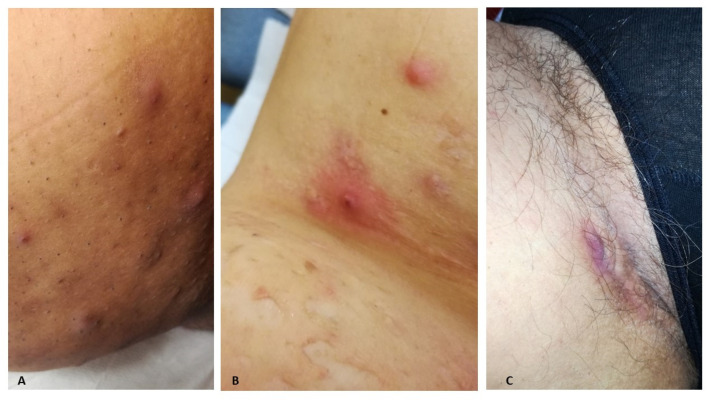
Typical clinical presentation of HS lesions: (**A**) Multiple inflammatory nodules located in the right groin region of a young woman, with dilated open comedones. Patient presented other nodules on the left side, 2 abscesses in the pubic region (Hurley score II, IHS4 16), and a mild form of papulo-pustular acne of the face. Diagnosis of HS was performed within one year of the first lesions’ development at the age of 15. (**B**) Abscess located in the axillary region of a 55-year-old female affected by severe HS since the age of 23. Scarring areas surrounded active lesions. Hurley III score and IHS4 of 20. Lesions were associated with VAS pain of 8 (0–10 range). (**C**) Draining tunnel located at the right inguinal region of a 50-year-old patient with a long history of HS since the age of 14. Lesions were underestimated by the patient and a diagnostic delay of more than 20 years occurred just after the development of severe cutaneous psoriasis.

**Figure 2:**
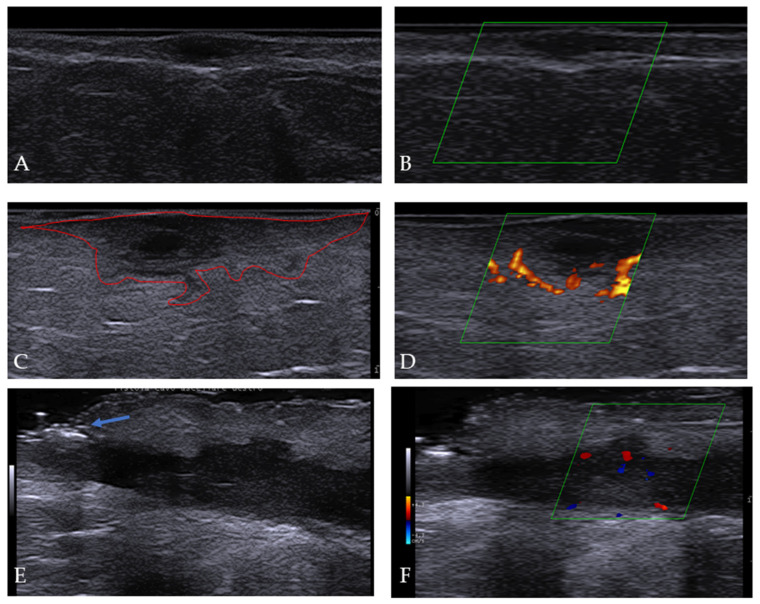
Cutaneous scans of areas affected by HS with 18 mHz probe revealed the presence of: (**A**) A nodule that presented as a round anechoic nodular dermal lesion. (**B**) Absence of power doppler signal as in a non-inflammatory nodule. The lesion was the result of a treated abscess with topical clindamycin and it was stable through 12 weeks of follow-up with twice-weekly topical clindamycin application. Lesion was surgically excised thereafter. (**C**) Abscess displaying as a hypoechoic/anechoic fluid deposit with a polypoid aspect and connection to the base of a widened hair follicle. (**D**) Power doppler study revealed a peripheral signal corresponding to the polypoid extension of the periphery of the lesion. (**E**) A tunnel characterized by an anechoic/ipoechoic band-like structure in the dermis/hypodermis and connected to the base of a widened hair follicle (blue arrow). (**F**) Peri-intra power doppler signal of the tunnel.

At the moment of our first observation, patients presented a median number of three body sites involved in HS (IQR: 2–5): the inguinal (34/53 patients, 64.2%), and axillary (28/53 patients, 52.8%) regions were the most frequently involved areas, followed by the anogenital (18/53 patients, 34%) and gluteal (16/53 patients, 30.2%) areas. The trunk (8/53 patients, 15.1%) was less commonly affected by HS, and lesions were all located in the sub-mammary/mammary area. Clinical and ultrasonographic assessment revealed the presence of nodules in 35/53 (66%) patients, abscesses in 39/53 (73.6%) patients, and tunnels in 29/53 (54.7%) patients (Figure 1 and Figure 2).

### 3.2. Patients with Early- vs. Adult-Onset HS

#### Clinical Features

We found that early-onset HS was not uncommon, with 22/53 (41.5%) patients developing the disease below the age of 18 years (median age of onset: 14 years; IQR 11–16).

Although the average age at our first observation was different between the two groups (26 years, IQR 18–30 for early-onset vs. 32 years, IQR 27–49 for adult-onset HS patients), no differences in the overall duration of the disease (11 years, IQR 5–22 for early-onset vs. 4 years, IQR 3–15 for adult-onset HS patients; *p* = 0.114), as well as in the diagnostic delay (6 years, IQR 2–9.5 for early-onset vs. 2 years, IQR 1–6 for adult-onset HS patients; *p* = 0.156) were observed. These data helped us to remove any possible bias regarding differences in the severity of the disease due to therapeutic delay and worsening of the disease.

Patients with early-onset HS had a significantly lower age of first menstrual cycle occurrence (11.5 years; IQR 10–13 for early-onset versus 13, IQR 12–13.8 for adult-onset HS patients; *p* = 0.0226), had significantly more frequent menstrual cycle abnormalities (9/22 (40.9%) early-onset-HS versus 4/31 (12.9%) adult-onset HS patients; *p* = 0.0264). Three of twenty-two (13.6%) early-onset patients had PCOS (versus 1/31 adult-onset HS patient, 3.2%), two of twenty-two (9.1%) and zero of thirty-one (0%) patients had precocious puberty, while one of the thirty-one (3.2%) adult-onset patients was under investigation for suspected endometriosis. In 4/22 (18.2%) early-onset patients, the onset of HS preceded puberty. 

A great proportion (14/22, 63.3%) of patients with early-onset HS had BMI > 25. Overweightness was also observed in 50% of patients with an age at visit below 20, all belonging to the early-onset HS group. 

The average BMI in early- and adult-onset HS was similar, and the global percentage of HS patients with BMI > 25 was greater than the percentage of Italian over-weight women (52%) [19], with special regard to early-onset HS patients. 

Smoking habits or personal history of acne were not related to early-onset HS (*p* = 0.95 and *p* > 0.9999, respectively).

No differences in the Hurley and IHS4 scores were observed between the two groups (*p* = 0.24 and 0.35 respectively), as well as in the number of affected areas (*p* = 0.09). The two groups also displayed similar HS morphology, with no differences in the number of nodules, abscesses, or tunnels.

**Figure 3:**
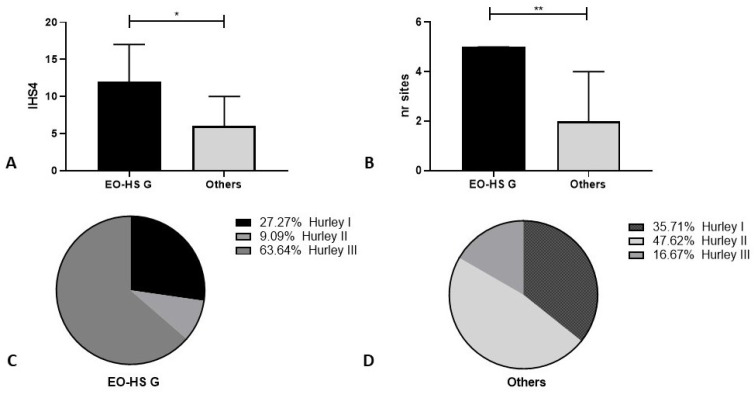
Disease severity was scored according to the Hurley classification, IHS4 scores, and widespread distribution of lesions. (**A**) IHS4 scores (median, IQR) were significantly higher in patients with early-onset HS and genital localization of lesions (EO-HS G; black bar) compared with all the other (others: grey bar) patients (*p* = 0.0242, *); (**B**) patients in the EO-HS G group (black bar) had an increased number of body sites (nr sites) involved in HS compared with other patients (others; grey bar) (*p* = 0.0042, **); Hurley scores of EO-HS G (**C**) were significantly higher compared with all the other patients (**D**) (*p* = 0.03; X2 trend). The graphs show the distribution of Hurley scores among patients in the two studied groups.

The ano-genital area was more frequently affected in patients with earlier onset of the disease (11/22, 50%) versus patients with adult-onset (7/31, 22.6%) disease (*p* = 0.0459). The ano-genital area’s involvement in the early-onset-HS group was also a negative factor in the global severity of the disease: these patients had significantly higher Hurley (*p* = 0.0344) and IHS4 scores (*p* = 0.0242) and a higher number of affected areas compared with all of the other patients studied (*p* = 0.0042) (Figure 3).

### 3.3. Treatment Approach

Overall, almost 25% of patients were naïve to any systemic treatment. All of the other patients had been previously treated with oral corticosteroids (3/53, 5.7% patients), acitretin (2/53, 3.8% patients), isotretinoin (2/53, 3.8% patients), oral antibiotics (oral clindamycin, followed by azitromycin, tetracyclin, minociclin; 37/53, 69.8% patients), and/or biologic agents (10/53, 18.9% patients). Only 5/53 (9.4%) patients had already undergone surgery.

At our first observation, we prescribed only a topical agent to 28/53 (52.8%) patients, conventional systemic treatments (antibiotics and/or acitretin) to 14/53 patients (26.4%), and biologics (including adalimumab, infliximab, and secukinumab) to 11/53 patients (20.8%). 

During the visit, patients were invited to reduce/discontinue smoking and to modify their dietary milk and complex carbohydrates intake [20]. When possible and needed, a weight loss program was considered as part of the treatment. Patients were also instructed regarding the correct use of topical agents, and help with medications was provided. According to our treatment protocols, almost all the patients applied topical clindamycin, not only during flares, but also twice/weekly as maintenance. Moreover, almost all patients were invited to take food supplements variously containing myo-inositol, zinc, biotin, plant sterols, and lactic ferments [21,22]. These data might account for the smaller number of patients who were considered candidates for systemic therapy (25/53, 47.2%). In five cases, surgery was the selected therapeutic option, either alone or in combination with treatment per os. Early-onset HS patients (8/22, 36.4% versus 3/31, 9.7% adult-onset HS patients) were more frequently treated with biologic agents (*p* = 0.0486).

### 3.4. Ultrasonographic Features

Early- and adult-onset HS patients shared common typical US features of HS, including nodules, abscesses, tunnels, dermal thickening, and retained hair tracts [23,24] (Figure 3). In many cases, underlying abscesses or tunnels were identified with US, allowing a higher accuracy of stadiation, as previously reported by Wortsman X and colleagues [25]. For this reason, the IHS4 score was calculated by merging both clinical and US data. A power doppler study revealed that, when present, the signal was peri- and peri-intra-lesional, as previously observed [26].

We could not detect any differences in the morphology of the lesions, in SOS-HS scores, or in the power doppler signal between early-onset and adult-onset HS patients.

## 4. Discussion

Despite the increased interest in HS during the last decade, there is still a great discordance between the age of the first symptoms’ occurrence and the diagnosis, ranging from 7.2 up to 10 years, with negative social, psychological, and treatment burdens [2,26,27,28]. Recently, we and other authors have shown that a longer diagnostic delay is associated with more severe phenotypes of the disease, younger age of onset of HS, female sex, and a negative impact on treatment efficacy [6,26,29,30].

HS is characterized by a very high negative impact on quality of life, with a psychosocial burden and sexual impairment. These aspects are under investigation, and causes of higher frequency of sexual distress in women have been linked to anatomic localization (groin and genitals), intensity of pain, and unpleasant odor [3,31,32].

In our population, earlier onset of HS was characterized by a more frequent localization in the genital area. This feature also represented a negative factor in the global severity of the disease. Patients with both early-onset and genital HS had significantly higher Hurley and IHS4 scores as compared with patients with extragenital localization, which was also due to the higher number of affected areas. These data are in line with results we have previously published for patients of both sexes with pediatric-onset HS, who were aged under 18 years at the moment of our observation [29].

Bettoli V et al. [6] observed that, while they could not detect differences in the frequency of axillary, inguinal/crural area localization or in the severity of disease evaluated by the Sartorius score, gluteal/intergluteal and mammary body regions were more frequently involved in early-onset patients versus patients who developed HS after the age of 16 years. In our study, we could not confirm these findings, potentially due to the exclusion of males from our population. However, this observation might be reasonable if we look at our recently published data detecting an association with longer diagnostic delay, early onset, and posterior body sites’ involvement in HS lesions [29].

A previous study by Deckers IE et al. [13] reported widespread disease in patients with earlier development of lesions, consistent with our findings in the current study and our previous findings [29].

We also found that patients with early-onset HS had a significantly lower age of first menstrual cycle occurrence. Association with PCOS and HS has been reported in 4% to 12.5% of cases [33,34], while pediatric HS has been suggested as a possible marker of precocious puberty [35,36]. Offidani A et al. [13] discussed eight cases with prepubertal onset, highlighting that there is a need for the multidisciplinary management of those patients with regard to associated precocious puberty, hyperinsulinism, and hyperandrogenism together with dedicated therapeutic guidelines. 

Personal history of acne was not related to early-onset HS, and, although acne conglobata was found to be a risk factor for the early development of HS [9], we should take into account that the population in this study was composed only of females. 

More than 50% of our patients were smokers, confirming the significant association between smoking habits and HS [37,38], although no differences were reported in the frequency of active or former smokers between early- and adult-onset HS. Smoking exposure during daily activities from housemates to the workplace was not investigated due to the retrospective nature of our study in order to avoid missing data; however, earlier exposure to passive smoking during childhood or adolescence has been reported as a trigger factor.

HS patients, particularly early-onset HS patients, were largely overweight. This, in accordance with the findings of other authors, might suggest that a higher BMI could be associated with earlier onset of HS [39]. Despite our data not revealing worse prognosis in obese patients, we firmly recommended weight loss and a reduction in dietary milk products and carbohydrates, as suggested in the literature, to reduce general inflammation and friction stimuli. Food supplements enriched in zinc, being aware of interactions with minociclin, or inositol might play a role in skin appendages’ activity and in PCOS or insulin resistance modulation. Available food supplements often combine all these elements also with probiotics, and this could be an advantage for skin microbiota. Although only half of the patients were candidates for systemic therapy, early-onset HS patients required, more frequently, the use of biologic agents in order to obtain clinical benefits, confirming that this subset of patients had a more complex disease and required a different therapeutic approach [13,15]. The extensive use of topical and systemic antibiotics causes treatment failure over time and, according to previous studies, the formation of drug-resistant strains in colonized HS lesions [40,41,42,43]. For this reason, as a general rule, we also suggest the early introduction of biologic therapy in patients with Hurley II or more than two involved areas as a disease modulator. The detection of a power/color doppler signal at ultrasonography examination that indicated vascularization and inflammation of the lesions, especially at the periphery of abscesses, where it could suggest initial tunnel development, can be also considered as a treatment selection criterion. The doppler signal could be a useful tool for introducing adjuvant systemic treatment to reduce the activity and dimensions of nodules, abscesses, and tunnels before surgery and to identify predictive failure markers of biologic therapies [44,45,46,47]. The use of other techniques in diagnosis and monitoring treatment, such as LASCA (laser speckle contrast analysis) or OCT (optical coherence tomography), has been recently reported, highlighting the importance of new tool development for HS [48,49]. Complementary treatments, including photodynamic therapy (PDT) and more recent surgery approaches, should also be considered [50,51,52]. Clinicians should integrate different techniques and combine medical and surgical protocols, allowing a decrease in antibiotics use and an increase in disease control over time. 

Finally, although there are no established clinical predictors of disease severity, earlier onset of disease and disease duration have been suggested as negative prognostic factors, and prompt intervention is highly recommended to limit disease evolution.

## 5. Conclusions

In our study, we showed that a younger age at the presentation of the disease might impact the clinical phenotype of HS, with a prevalent genital localization and more severe and disseminated disease, and the treatment choices. These data are relevant for reducing the diagnostic gap between the first symptoms’ onset and diagnosis, providing adequate psychological support, and establishing tailored treatment protocols.

## Figures and Tables

**Table 1:** Clinical and demographic features of female patients affected by HS. HS severity (Hurley and HIS4), morphology, and treatment. The table shows data on the overall population (All) and early-onset HS (<18 years) versus adult-onset HS (≥18 years) patients.

	All (n = 53)	Early Onset (n = 22)	Adult Onset (n = 31)	*p*
**Age at visit, years °**	30 (22–43)	26 (18–30)	32 (27–49)	0.0005 §
**Age of onset, years °**	19 (14–25)	14 (11–16)	25 (20–34.2)	<0.0001 §
**Age at diagnosis, years °**	25 (20–35)	18 (14–24)	27 (22.8–43)	<0.0001 §
**HS duration, years °**	7 (4–16)	11 (5–22)	4 (3–15)	0.11 §
**Delay in diagnosis, years °**	3.5 (1–9)	6 (2–9.5)	2 (1–6)	0.16 §
**Family history, n (%)**	7 (13.2)	5 (22.7)	2 (6.5)	0.11 #
**Smoking, n (%)**				
Never smoked	17 (32)	7 (31.8)	10 (32.3)	
Former smokers	4 (7.5)	2 (9.1)	2 (6.3)	
Active smokers	32 (60.4)	13 (59.1)	19 (61.3)	0.95 *
**Personal history acne, n (%)**	15 (28.3)	6 (27.3)	9 (29)	>0.9999 #
**First menstruation age, n (%)**	12 (11–13)	11.5 (10–13)	13 (12–13.8)	0.0226 §
**Menses abnormalities, n (%)**	13 (24.5)	9 (40.9)	4 (12.9)	0.0264 #
**BMI kg/m^2^ °**	25.2 (21.3–28.5)	25.7 (23.5–29.8)	25.2 (20.8–28.3)	0.57 §
BMI > 25, n (%)	31 (58.5)	14 (63.3)	17 (54.8)	0.58 #
BMI > 30, n (%)	11 (20.8)	4 (18.2)	7 (22.6)	0.75 #

° median, IQR § Mann–Whitney test, # Fisher’s exact test, * X2 trend.

**Table 2:** HS severity (Hurley and HIS4), morphology, and treatment. The table shows data on the overall population (ALL) and early-onset HS (<18 years) versus adult-onset HS (≥18 years) patients.

	All (n = 53)	Early Onset (n = 22)	Adult Onset (n = 31)	*P*
**Hurley, n (%)**				
**I**	18 (33.4)	5 (22.7)	13 (41.9)	
**II**	21 (39.6)	9 (40.9)	12 (38.7)	
**III**	14 (26.4)	8 (36.3)	6 (19.4)	0.24 *
**IHS4 °**	6 (3.3–11.8)	8.5 (3.8–12)	5.5 (3–10)	0.35 §
**Region involved in HS (n)**				
Axillae	28 (52.8)	12 (54.5)	16 (51.6)	>0.9999 #
Inguinal	34 (64.2)	17 (77.3)	17 (54.8)	0.15 #
Gluteal	16 (31.4)	7 (31.8)	9 (29)	>0.9999 #
Trunk	8 (15.1)	2 (9.1)	6 (19.4)	0.45 #
Ano-genital	18 (33.4)	11 (50)	7 (22.6)	0.0459 #
**Type of lesions (n)**				
Nodules	35 (66)	16 (72.7)	19 (61.3)	0.56 #
Abscesses	39 (73.6)	18 (81.8)	21 (67.7)	0.35 #
Fistulas/tunnel	29 (54.7)	14 (63.4)	15 (48.4)	0.40 #
**Number of lesions °**				
Nodules	1 (0–3)	1.5 (0–3)	1 (0–3)	0.73 §
Abscesses	1 (0–2)	1 (0.75–2.25)	1 (0–2)	0.86 §
Fistulas/tunnel	1 (0–1)	1 (0–1.25)	0 (0–1)	0.39 §
**Number of affected areas °**	3 (2–5)	3.5 (2–5)	2 (2–4)	0.09 §
**Previous HS treatments, n (%)**				
Only topical agents	13 (24.5)	6 (27.3)	7 (22.6)	0.75 #
Systemic agents				
*Conventional*	44 (83.1)	17 (77.3)	27 (87.1)	0.46 #
*Biologics*	10 (18.9)	6 (27.3)	4 (12.9)	0.29 #
**Actual HS treatment, n (%)**				
Only Topical agents	28 (52.8)	11 (50)	17 (45.9)	0.59 #
Systemic agents				
*Conventional*	14 (26.4)	3 (13.6)	11 (35.5)	0.11 #
*Biologics*	11 (20.8)	8 (36.4)	3 (9.7)	0.0486 #

° median, IQR § Mann–Whitney test, # Fisher’s exact test, * X2 trend.

## Data Availability

Data are available on request to the corresponding author.
